# Autosomal Dominant Calpainopathy in a Diabetic Patient Complicated by Functional Gitelman Syndrome

**DOI:** 10.1155/carm/4210190

**Published:** 2025-06-05

**Authors:** Kabilash Manivalli Peterpalaniswami, Krishnaswamy Madhavan, Gerry George Mathew, V. Jayaprakash

**Affiliations:** ^1^Department of General Medicine, SRM Medical College Hospital and Research Centre, Kattankulathur, Kanchipuram 603203, Tamil Nadu, India; ^2^Department of Nephrology, SRM Medical College Hospital and Research Centre, Kattankulathur, Kanchipuram 603203, Tamil Nadu, India

**Keywords:** autosomal dominant limb girdle muscular dystrophy, calpainopathy, Gitelman syndrome, hypokalemia, hypomagnesemia

## Abstract

Adult-onset Gitelman syndrome with calpainopathy is a rare clinical condition in patients with diabetes mellitus. We present the case of a 52-year-old male diabetic patient who presented with muscle weakness and fatigue. On evaluation, he had reduced power in the thigh and pelvic girdle muscles. Laboratory tests revealed hypokalemia, hypomagnesemia, metabolic alkalosis, kaliuresis, and hypocalciuria, which led to the diagnosis of Gitelman syndrome. Electromyography revealed a myopathic pattern with polyphasic motor unit action potentials of a short duration. Genetic analysis revealed a heterozygous mutation in CAPN3, suggestive of autosomal dominant calpainopathy or limb girdle muscular dystrophy. He was administered intravenous potassium and magnesium supplements, followed by oral potassium chloride, magnesium oxide, and potassium-sparing diuretics. The patient had improved muscle strength on follow-up, with resolution of the electrolyte abnormalities. This case report highlights this rare clinical entity, its variable clinical manifestations, and the pathophysiological mechanisms involved in electrolyte abnormalities.

## 1. Introduction

Gitelman syndrome is a rare autosomal recessive renal tubulopathy characterized by metabolic alkalosis, hypokalemia, normotension, hypomagnesemia, and hypocalciuria [1]. It is generally attributed to inactivating mutations in SLC12A3, which encodes thiazide-sensitive sodium chloride channels in the distal convoluted tubule (DCT) [1]. Calpainopathy is a group of heterogeneous disorders characterized by limb girdle muscular dystrophy due to mutations in the gene encoding the proteolytic enzyme calpain 3 [2]. Here, we describe a rare association between Gitelman syndrome and calpainopathy and the possible pathophysiological mechanisms governing this rare clinical consortium.

## 2. Case Report

**Figure 1:**
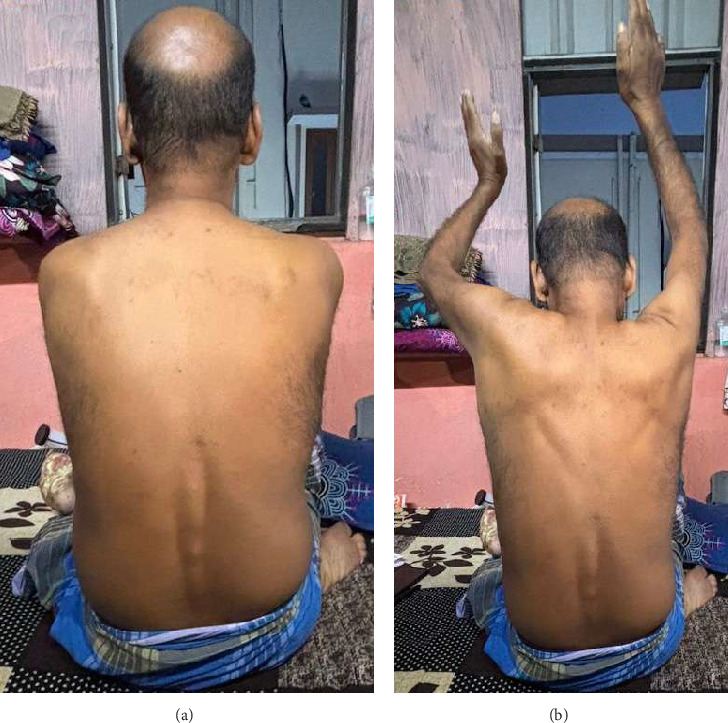
(a) Clinical picture of the patient demonstrating mild drooping of the left shoulder compared with the right shoulder (magnification: 600 DPI). (b) Clinical picture of the patient demonstrating difficulty in overhead abduction bilaterally indicating weakness of deltoids, supraspinatus, infraspinatus, subscapularis, and serratus anterior (magnification: 600 DPI).

A 52-year-old brittle diabetic patient with diabetic neuropathy and retinopathy presented to the hospital with new-onset difficulty in climbing stairs, myalgia of thigh muscles, back pain, and bilateral upper shoulder pain with drooping (Figures 1(a) and 1(b)) since the last 6 months. Patient was on gliclazide 80 mg/day, metformin 1500 mg/day, dapagliflozin 10 mg/day, and sitagliptin 100 mg/day for diabetes mellitus. On clinical evaluation, the patient was normotensive with a blood pressure of 110/70 mmHg and reduced power of 4/5 at the level of the thighs, shoulder, and pelvic girdle muscles for all ranges of motion in addition to reduced vibration and position sense, which was attributed to his existing diabetic neuropathy and amyotrophy. The patient did not have any salt craving or polyuria. Laboratory investigations revealed random blood sugar: 259 mg/dl, creatinine: 1.3 mg/dl, urea: 37 mg/dl, sodium: 134 meq/litre, k=2.4 meq/l (reference: 3.5-5 meq/litre), cl: 93 meq/litre, bicarbonate: 32 meq/litre, magnesium: 1.3 mg/dl (reference: 1.7-2.2 mg/dl), calcium: 8.2 mg/dl, phosphorous: 2.6 mg/dl, haemoglobin: 9.9 grams/dl, total creatinine kinas: 145 IU/L and normal bilirubin with normal liver enzyme levels. Urine examination revealed bland urine sediments without albuminuria. Arterial blood gas analysis revealed a pH of 7.45, PCO_2_ of 40 mmHg, and bicarbonate level of 34 mEq/L, suggestive of metabolic alkalosis. There were no incriminating drugs or clinical conditions attributable to metabolic alkalosis, hypokalemia, or hypomagnesemia in this patient on history and clinical evaluation. The urine spot potassium creatinine ratio was 3.96 mmol/mmol (> 1.5 mmol/mmol—urinary potassium loss), and the urine spot calcium creatinine ratio was 0.15 mg/mg (> 0.2 mg/mg—urinary calcium loss) which was suggestive of kaliuresis and hypocalciuria. In the context of normotension, metabolic alkalosis, hypokalemia, hypomagnesemia, kaliuresis, and hypocalciuria, a clinical diagnosis of Gitelman syndrome was made. In view of the reduced proximal muscle power, an electromyogram was performed, which was suggestive of a myopathic pattern with polyphasic motor unit action potentials of shorter duration. Nerve conduction studies revealed reduced sensory nerve action potential amplitudes and sensory nerve conduction velocity, suggestive of diabetic peripheral neuropathy. Clinical exome analysis was performed for late-onset Gitelman syndrome, which incidentally revealed a germline heterozygous mutation in CAPN3 (CAPN3(NM_000070.3):c.1466G > A; p.(Arg489Gln)) responsible for autosomal dominant limb girdle muscular dystrophy. There was no obvious mutation of SLC12A3 on whole exome sequencing (WES). Muscle biopsy was not attempted because the patient did not provide consent. He was administered intravenous potassium chloride and magnesium supplementation, followed by oral potassium chloride and magnesium oxide supplementation along with spironolactone. On follow up after 15 days, his serum potassium was 3.7 meq/litre and magnesium–1.9 meq/litre on oral potassium chloride (60 meq/day) and magnesium oxide (1200 mg/day) with improved muscle strength and mobility.

## 3. Discussion

Gitelman syndrome is a familial autosomal recessive disorder characterized by metabolic alkalosis, hypocalciuria, normotension, and renal wasting of potassium and magnesium due to defects in *SLC12A3* [1, 2]. This syndrome is clinically characterized by muscle weakness, failure to thrive, and fatiguability and is predominantly a disease of the young [1, 3]. Adult-onset Gitelman syndrome is rare and is usually a result of a compound heterozygous mutation in the *SLC12A3 gene,* which results in loss of function of the gene [3]. Missense mutations account for 70% of all mutations, and there is increased susceptibility to large rearrangements caused by repeated sequences within the incriminating gene in adult-onset Gitelman syndrome [3]. Chronic hypokalemia and hypomagnesemia in this syndrome can hinder glucose metabolism, insulin sensitivity, and secretion in diabetic patients, which can predispose them to brittle diabetes with microvascular complications, as observed in our patient [4].

Autosomal dominant calpainopathy or limb girdle muscular dystrophy is a mild variant of the calpain 3 gene mutation mapped to chromosome 15 and presents in adulthood with involvement of pelvic girdle muscles, thigh, and axial muscles [5]. There is significant intrafamilial and clinical variability in the presentation of autosomal dominant calpainopathy [2, 5]. Back pain and hyperlordosis along with involvement of the thigh and shoulder muscles are some of the clinical features of calpainopathy [2, 5]. The challenge in the diagnosis of autosomal dominant calpainopathy is the subtle involvement of muscle groups, normal creatinine kinase levels, and minimal changes in muscle biopsy [5]. Our patient had mildly reduced power in the thigh and pelvic girdle muscles and normal creatinine kinase levels in the background of diabetes mellitus, which confounded our initial differential diagnosis as diabetic amyotrophy.

Calpain 3 belongs to a superfamily of nonlysosomal cysteine proteases which have relevant cell motility, cell differentiation, apoptosis, and cell-cycle regulation functions [6]. CAPN3 mutation leads to defective function of the sodium calcium exchanger (NCX) at the basolateral aspect of DCT cells, which leads to increased calcium retention in the intracellular compartment [6]. Calcium dysregulation along with defective calcium/calmodulin-dependent protein kinase II in this mutation leads to intracellular calcium balance dysregulation along with impaired gene transcription, oxidative stress, and mitochondrial abnormalities [6]. Calcium dyshomeostasis with defective gene transcription hampers the calcium concentration microdomains in the cytosol, which ultimately leads to the suboptimal function of sodium potassium ATPase pump (Na-K ATPase) at the basolateral aspect of DCT cells [7]. An appropriate physiological cytosolic calcium concentration is required to improve the sensitivity of potassium binding to the Na-K ATPase pump, which is hampered by the CAPN3+ mutation [6–8]. We postulate that a defective NCX and sarcoplasmic/endoplasmic reticulum calcium ATPase (SERCA) pump in CAPN3 mutation lead to increased intracytosolic calcium levels, thereby leading to calcium-sodium competition for binding sites on the Na-K ATPase pump [6–8]. This leads to the suboptimal functioning of Na-K ATPase, resulting in intracellular sodium retention and defective absorption of sodium through the sodium chloride (Na-Cl) channel due to the disrupted concentration gradient. Loss of the sodium concentration gradient leads to functional Gitelman syndrome due to impaired functioning of the apical thiazide-sensitive Na-Cl channel. The connecting tubules absorb the remnant sodium load, thereby translating to potassium and hydrogen loss in the urine by downstream mechanisms culminating in hypokalemia and metabolic alkalosis [7].

The expression of the transient receptor potential cation channel subfamily M 6 (TRPM6) channel on DCT is governed by the extracellular signal–regulated kinase (ERK) limb of the mitogen-activated protein kinase (MAPK) superfamily, protein kinase C (PKC), phosphoinositide 3-kinase (PI3K), protein kinase A (PKA), and phospholipase C and D pathways [9]. In calpainopathy, there is a defective function of PI3K, which may result in decreased expression of TRPM6, leading to hypomagnesemia [6, 10]. The functional defect of the thiazide-sensitive Na-Cl channel results in the shortening of the segment, reducing the Mg^2+^ reabsorption capacity [10]. The basolateral membrane potential, governed by Na^+^ -K^+^ -ATPase activity, is impaired in calpainopathy, which further hinders magnesium extrusion [11]. The hypocalciuria in our patient is probably related to enhanced calcium absorption in the proximal tubule due to the relative hypovolemia generated by this functional Gitelman syndrome [1, 2].

The conventional management of autosomal dominant calpainopathy is purely conservative, with most patients becoming wheelchair-bound by the age of 60 [2, 5]. Treatment modalities encompass physical therapy and stretching exercises to enhance mobility, mobility aids such as canes, walkers, orthotics, and wheelchairs to facilitate independence, supervised strengthening and gentle low-impact aerobic exercise, nutritional management, knee-ankle-foot orthoses during sleep to prevent contractures, surgical intervention for foot deformities and Achilles tendon contractures as necessary, annual influenza vaccination, prompt treatment of chest and respiratory infections, nocturnal ventilator assistance as required, respiratory aids to address chronic respiratory insufficiency in advanced stages of the disease, and social, emotional, and familial support for care-related decision making [2, 5]. The therapeutic approach for Gitelman syndrome centers on the judicious correction of electrolyte imbalances, specifically hypokalemia and hypomagnesemia, through targeted supplementation [1]. This regimen may be augmented with the administration of potassium-sparing diuretics, angiotensin receptor antagonists, or nonsteroidal anti-inflammatory compounds such as indomethacin, based on the patient's clinical presentation and requirements [1].

The limitation of this case study is the possibility of nondetection of a pathogenic mutation in the second CAPN3 allele and intronic splicing variants in WES. Another pertinent limitation is lack of DNA samples from other family members to establish the autosomal dominant nature of this mutation. Even deep intronic mutations in SLC12A3 which is responsible for some variants of Gitelman syndrome cannot be detected by WES. However, this case highlights a rare association of possible functional Gitelman syndrome with autosomal dominant calpainopathy and opens research avenues to explore the intricate molecular interactions of calpain 3 with various transport channels in the renal tubules.

## Data Availability

The data that support the findings of this study are available on request from the corresponding author. The data are not publicly available due to privacy or ethical restrictions.
